# Metformin and Severe Post-COVID-19 Outcomes Among Individuals with Diabetes Mellitus

**DOI:** 10.64898/2026.07.06.26357398

**Published:** 2026-07-09

**Authors:** Zachary Butzin-Dozier, Lin-Chiun Wang, Yunwen Ji, A. Jerrod Anzalone, Oluwasolape Olawore, Ryan Hafen, Eric Hurwitz, Manav Kumar, Rena C. Patel, Ariana Budhihartanto, Mark van der Laan, John M. Colford, Alan E. Hubbard, John B. Buse, Steven Johnson, Jane Reusch, Lauren E. Chan, Richard Moffitt, Rachel Wong, Carolyn Bramante

**Affiliations:** 1 Stanford University School of Medicine, Stanford, CA, USA; 2 School of Public Health, University of California, Berkeley, Berkeley, CA USA; 3 University of Nebraska Medical Center, Omaha, NE, USA; 4 University of North Carolina at Chapel Hill, Chapel Hill, NC, USA; 5 Purdue University, West Lafayette, IN, USA; 6 University of Alabama at Birmingham, Birmingham, AL, USA; 7 University of Minnesota, Minneapolis, MN, USA; 8 University of Colorado, Anschutz, Aurora, CO, USA; 9 University of Chicago, Chicago, IL, USA; 10 Renaissance School of Medicine, Stony Brook University, New York, NY, USA

## Abstract

**Background:**

Metformin is one of the most commonly prescribed medications for individuals with diabetes and may provide protection against long-term sequelae of COVID-19.

**Methods:**

We evaluated a retrospective cohort of individuals in the National Clinical Cohort Collaborative with type 2 diabetes mellitus and COVID-19 who were prescribed metformin or a dipeptidyl peptidase-4 inhibitor (DPP4i) at least 30 days before the onset of acute COVID-19 between October 1, 2021, and November 15, 2023. We compared the 12-month cumulative incidence of Long COVID diagnosis (ICD-10 U09.9: Post COVID-19 condition, unspecified), probable Long COVID (based on a model-derived phenotype), and mortality between individuals prescribed metformin vs. DPP4i. We applied Super Learner and targeted maximum likelihood estimation to obtain risk ratios while adjusting for covariates of interest.

**Results:**

In our sample of 53,332 individuals with type 2 diabetes and COVID-19, we found that metformin prescription was associated with a lower risk of all-cause mortality after COVID-19 (adjusted risk ratio [aRR] 0.61, 95% CI 0.51, 0.73). We also observed that metformin users, compared to DPP4i users, had a slightly lower risk of probable Long COVID (aRR 0.87, 95% CI 0.81, 0.94) but did not detect a significant relationship with Long COVID diagnosis (aRR 0.90, 95% CI 0.68, 1.20), although we observed similar point estimates across Long COVID outcomes.

**Conclusions:**

These findings support the hypothesis that metformin prescription during acute COVID-19 may be associated with lower mortality among adults with diabetes. These analyses also provide modest evidence of a protective association against Long COVID in adults with diabetes, although estimates were imprecise.

Long COVID, also known as post-acute sequelae of COVID-19 or long-haul COVID, is a broad array of conditions and symptoms that individuals develop after the initial SARS-CoV-2 infection subsides.^1–3^ Individuals with diabetes are at increased risk of mortality after respiratory virus infections, and of post-infectious syndromes like Long COVID.^4–6^ Type 2 diabetes mellitus (T2DM) results from a combination of ineffective insulin secretion by pancreatic β-cells and reduced insulin sensitivity, and it is one of the most common metabolic disorders worldwide.^7,8^ Metformin, a first-line treatment for T2DM, is an antihyperglycemic medication with considerable immunomodulatory effects and host-mediated antiviral properties that may protect individuals against the long-term sequelae of COVID-19.^9–13^ Evaluating the relationship between antihyperglycemic medication use and severe post-COVID-19 outcomes may inform clinical management strategies for individuals with T2DM, a population at elevated risk for severe COVID-19 complications.

Randomized controlled trials have evaluated the impact of metformin on COVID-19 severity and the incidence of Long COVID.^9,14,15^ The COVID-OUT trial found that outpatient metformin use during acute SARS-CoV-2 infection was associated with lower odds (odds ratio 0.58, 95% confidence interval (CI) 0.35 to 0.94) of emergency department visit, hospitalization, or death, and 0.59 times the hazard (95% CI 0.39 to 0.89) of Long COVID.^11,15^ The ACTIV-6 randomized controlled trial found modest evidence that assignment to metformin, versus placebo, was associated with a decreased risk of Long COVID diagnosis over 6 months (adjusted risk ratio 0.50, 95% credible interval 0.16 to 1.00), but was not associated with increased Long COVID symptoms (adjusted risk ratio 0.79, 95% credible interval 0.47 to 1.23).^10^ Observational studies of individuals in the National Clinical Cohort Collaborative (N3C) have provided additional insights. Metformin was associated with a lower risk of mortality, hospitalization, and mechanical ventilation in individuals with T2DM and acute COVID-19.^16^ In a cohort of individuals with prediabetes, investigators found that metformin use, compared with levothyroxine or ondansetron, was associated with decreased acute COVID-19 severity as well as decreased mortality.^9^ In a cohort of individuals with T2DM, investigators found that metformin use, compared to sulfonylureas (SU) use, at the time of acute COVID-19 was associated with 0.56 times the risk of mortality (95% CI 0.33 to 0.97).^14^ Cumulatively, these results support the hypothesis that metformin use may reduce severe sequelae of COVID-19, such as Long COVID and mortality, although additional research is needed.

While previous studies have provided considerable evidence regarding the impact of metformin on acute COVID-19 and Long COVID, several questions remain. Randomized trials often suffer from poor generalizability, as trial populations often differ from the general population. In a cohort of individuals with T2DM in N3C, we aim to evaluate whether the prescription of metformin is associated with a lower risk of Long COVID and mortality, compared with the prescription of dipeptidyl peptidase-4 inhibitors (DPP4i). In two secondary cohorts (see Supplemental Material 1 for rationale), we evaluated the relationships between metformin, Long COVID, and mortality in individuals with (1) PCOS and (2) prediabetes to evaluate the generalizability of our findings in individuals with insulin resistance.

## METHODS

### Data source:

We analyzed data from the N3C, the largest open, national source of health data in U.S. history, containing data on 21 million individuals from 82 data providers.^17,18^ N3C includes electronic medical record data from participants ranging from January 2018 to the present.

### Sample:

We included individuals in N3C with type 2 diabetes mellitus (T2DM) aged 30–85 years who were diagnosed with COVID-19 between October 1, 2021 (the release date of the ICD code U09.9, Post COVID-19 condition, unspecified) and November 15, 2023, and were prescribed either metformin or DPP4i during (prevalent use) acute COVID-19 (metformin and DPP4i will hereafter be referred to as “study drugs”).^14^ We excluded individuals who (A) were first prescribed a study drug less than 30 days before acute COVID-19, (B) were prescribed multiple study drugs within 30 days before acute COVID-19, or (C) had a prescription end date before acute COVID-19. We did not exclude patients based on medication history more than 30 days before acute COVID-19, and we did not incorporate treatment change after acute COVID-19, following an emulated intention-to-treat design. The T2DM cohort included individuals with either a diagnosis code for T2DM or a hemoglobin A1C (HbA1C) greater than 6.5%. We excluded individuals diagnosed with any of the following conditions at baseline: chronic kidney disease stages 3, 4, or 5; end-stage renal disease; prediabetes (analyzed separately as a secondary prediabetes cohort); or polycystic ovarian syndrome (analyzed separately as a secondary PCOS cohort).^14^ We excluded individuals who were prescribed one of the study drugs as treatment for severe acute COVID-19 to avoid confounding by indication.

### Exposure:

The exposure of interest was the prescription of metformin compared to DPP4i, which is an alternative or adjunct pharmacotherapy to metformin and which has been demonstrated to have some reduction in COVID-19 severity.^14^

### Outcomes:

The outcomes of interest were the cumulative incidence of a Long COVID diagnosis (LC-Dx (defined by ICD-10 code U09.9: Post COVID-19 condition, unspecified), probable Long COVID (LC-P), or mortality within 12 months following the prescription of diabetes treatment medication. The outcome of interest, Long COVID, was defined using 2 different methods: 1) LC-Dx: diagnosis code U09.9, which became available in 10/2021 (LC-Dx), and to address limited capture of the Long COVID due to the poor early update of diagnosis code use 2) LC-P: a validated model-based phenotype based on patient conditions, symptoms, diagnoses, and laboratory measures.^19–23^ We considered a patient as having “probable Long COVID” if the patient had a Long COVID model-based phenotype score at or above 0.9 in 1–12 months following acute COVID-19. Previous studies have used 0.9 as a threshold for LC-P using the N3C Long COVID model-based phenotype.^24,25^

### Confounders and other covariates:

We included the following individual baseline covariates in our analyses based on potential relationships with the exposures and outcomes of interest (where the baseline is prior to prescription of study drug): healthcare utilization rate (healthcare interactions per month), sex, age, race and ethnicity, data provider, body mass index, tobacco use, medical conditions (obesity, chronic lung disease, hypertension, asthma, heart failure, dementia, arthritis, coronary artery disease, cancer, liver disease, chronic kidney disease, peripheral vascular disease, cerebrovascular disease, polycystic ovarian syndrome (PCOS), and depression), medication use (systemic corticosteroids, outpatient insulin, angiotensin converting enzyme (ACE) inhibitors, angiotensin receptor blockers, statins, anticoagulants, aspirin, torsemide, and furosemide), biomarker measurements (glycated hemoglobin A1c (A1c), serum creatinine, urine albumin to creatinine ratio, and estimated glomerular filtration rate [reported by EHR laboratory measurements]), and we tracked patient monitoring (frequency of healthcare interactions during baseline and follow-up period) (detailed covariate definitions published previously,^26^ covariate list included in Supplemental Material 2. For medical conditions and medication use, we evaluated any history between January 2018 (beginning of N3C observation period) and acute COVID-19. For biomarker measurements and BMI, we included the most recent value before acute COVID-19. We intervened on monitoring to evaluate the counterfactual impact of the treatment of interest, given that all individuals are monitored (healthcare interaction) once every six months during the study period.

### Secondary cohort:

As a secondary research question, we evaluated the relationship between metformin use, compared to levothyroxine, and Long COVID or mortality in cohorts of individuals with (A) prediabetes and (B) PCOS (excluding individuals with both prediabetes and PCOS). We selected levothyroxine as a comparator for our secondary cohort as it is a commonly prescribed oral medication (i.e., requires healthcare utilization) with no known antihyperglycemic or protective effects against COVID-19.^9,14^

### Analysis:

Our analysis applied Super Learner to maximize the prediction of the outcomes of interest, Long COVID and mortality, given individual treatment and covariate status, as well as the treatment mechanism (probability of prescription to metformin, given covariate information) and censoring mechanism (probability of loss to follow-up, given covariate and treatment information).^27–29^ Super Learner creates a convex combination of candidate algorithms and is guaranteed to perform at least as well as the best-fitting candidate algorithm in large sample sizes. Super Learner is ideal for this data setting, as high-dimensional covariate information will inevitably lead to model misspecification in traditional parametric analyses. Next, we used targeted maximum likelihood estimation to reduce bias and estimate the risk ratio comparing metformin use with DPP4i use for the one-year cumulative incidence of LC-Dx, LC-P, and mortality.^30–34^ Targeted maximum likelihood estimation is a doubly robust method that yields consistent inferences as long as the outcome regression or the treatment mechanism is estimated consistently. In this data setting, where the treatment decisions regarding metformin vs. DPP4i are difficult to characterize, this approach provides additional robustness against near positivity violations compared with inverse probability of treatment weighting alone.^30–32,34^ We considered the observation (healthcare interaction) as informative censoring (i.e., the censoring mechanism), estimating the counterfactual impact of the exposure (metformin prescription) under a scenario of universal observation.^27^ This counterfactual intervention included ensuring at least one healthcare interaction during the 12-month follow-up period, and for Long COVID outcomes, no mortality during the follow-up period.

## RESULTS

We evaluated EHR data from 53,332 individuals with T2DM, including 50,965 individuals who were prescribed metformin during acute COVID-19 and 2,367 who were prescribed DPP4i during acute COVID-19 (Table 1). In our sample, 52% of metformin users were female, while 59% of DPP4i users were female. The average age of metformin users was 60 years, while the average age of DPP4i users was 64 years. The mean BMI for metformin users was 36, and for DPP4i users, 35. The average baseline HbA1c was 7.7% among metformin users and 7.9% among DPP4i users. Metformin users had an average of 2.6 interactions with healthcare providers per month, whereas DPP4i users had an average of 2.7 interactions per month.

**Figure 1.**
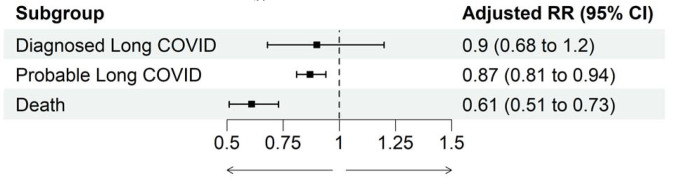
Adjusted relationships between metformin vs. DPP4i during acute COVID-19 and subsequent 12-month cumulative incidence of Long COVID or death, among individuals with type 2 diabetes mellitus.

We found that prescription of metformin, compared with DPP4i, during acute COVID-19 was associated with lower 12-month mortality risk (adjusted risk ratio (aRR) 0.61, 95% CI 0.51, 0.73; unadjusted risk ratio (uRR) 0.44, 95% CI 0.37, 0.53) and 12-month risk of LC-P (aRR 0.87, 95% CI 0.81, 0.94; uRR 0.85, 95% CI 0.78, 0.92) (Figure 1, Table 2). We did not find that prescription of metformin, compared to DPP4i, was significantly associated with 12-month cumulative incidence of Long COVID diagnosis (aRR 0.90, 95% CI 0.68, 1.20; uRR 0.80, 95% CI 0.57, 1.11)).

We evaluated EHR data from 15,582 individuals with prediabetes and 1,786 individuals with PCOS (see Supplemental Materials 3 and 4 for details on the prediabetes and PCOS cohorts). Among individuals with prediabetes or PCOS, we found that prescription of metformin, vs. levothyroxine, was not significantly associated with the one-year cumulative incidence of Long COVID diagnosis (prediabetes aRR 0.88, 95% CI 0.65 to 1.20; PCOS aRR 0.95, 95% CI 0.45 to 1.98), LC-P (prediabetes aRR 0.97, 95% CI 0.90 to 1.04; PCOS aRR 0.92, 95% CI 0.77 to 1.11), or mortality (prediabetes aRR 0.74, 95% CI 0.42 to 1.30 [PCOS invalid RR]) (see Supplemental Material 5).

## DISCUSSION

We found evidence that prescription of metformin, compared with DPP4i, was associated with lower mortality in T2DM individuals (aRR 0.61, 95% CI 0.51, 0.73). Our findings are consistent with the COVID-OUT trial, which randomized patients with COVID-19 to receive metformin, and found that metformin use during acute COVID-19 was associated with 0.58 times the odds (95% CI 0.35 to 0.94) of emergency department visit, hospitalization, or death. ^11^ Similarly, our findings were consistent with an observational study of individuals in N3C, which found that metformin use, compared to sulfonylureas, was associated with 0.56 times the risk of mortality (95% CI 0.33 to 0.97).^14^ The consistency of these findings across multiple study designs, including randomized trials and observational analyses using both prevalent and incident exposure definitions with different active comparators, supports the association between metformin use and lower mortality among individuals with T2DM and COVID-19. For discussion of secondary cohort findings, see Supplemental Material 6.

We found moderate evidence of a relationship between metformin prescription, compared to DPP4i prescription, and Long COVID among T2DM individuals. We observed similar point estimates for LC-Dx (aRR 0.90) and LC-P (aRR 0.87), although our estimate of LC-Dx had low precision due to LC-Dx being a rare outcome, with a cumulative incidence of 1% among individuals prescribed metformin and 2% among controls. This low diagnostic rate may be attributed to the wide range of Long COVID phenotypes or the lack of meaningful treatments for Long COVID, which may discourage formal diagnosis of the condition.^19,21,35^ Therefore, we included LC-P as an additional outcome, which uses a model-based phenotype and has greater sensitivity than LC-P. In contrast, LC-P may have lower clinical utility than LC-Dx, as it relies on myriad patient characteristics and conditions to approximate underlying disease status but lacks clear clinical application. The inclusion of both measures serves to evaluate the consistency of the relationships between these outcomes. Our observed point estimates were considerably more modest than previous studies, as the COVID-OUT trial found that assignment to metformin within 3 days of COVID-19 onset reduced the hazard of Long COVID (hazard ratio 0.59, 95% CI 0.39 to 0.89).^15^ The attenuated relationship observed in our study, compared to the COVID-OUT trial, may be due to residual confounding of the relationship between metformin prescription and LC-Dx and LC-P by comorbidity burden, differential monitoring, or healthcare utilization. These results add to the mixed findings of the cumulative literature, which have shown heterogeneous impacts of metformin on Long COVID depending on the definition of Long COVID used.^10,11,15^

Metformin may protect individuals with T2DM and acute COVID-19 from mortality and Long COVID through similar mechanisms. In addition to its antihyperglycemic effects, metformin has shown anti-inflammatory properties and modulates interleukin-6 and interleukin-1, which are key cytokines that trigger hyperinflammation in severe COVID-19 and Long COVID.^11,36–39^ Furthermore, metformin has demonstrated antiviral activity against SARS-CoV-2, which may improve recovery from COVID-19 and prevent Long COVID.^11,40,41^

### Strengths and Limitations

The major limitation of our study is that we did not address the effects of initiating these drug classes during the acute COVID infection, thus limiting the causal interpretation of our findings. The relatively small proportion (0.04) of T2DM individuals using DPP4i in our sample leads to imprecise estimates due to low power, particularly regarding rare outcomes like Long COVID diagnosis, which had only a 1% cumulative incidence over study period). Residual confounding by indication remains a concern, as metformin is considered a first-line treatment for T2DM patients, while DPP4i is typically a second-line or add-on therapy.^42,43^ Therefore, DPP4i patients may be less amenable to antihyperglycemic medications or may have advanced T2DM characteristics that are not captured in the EHR. The generalizability of the N3C sample is another limitation. While N3C is a large, national sample, it oversamples individuals from research institutions and individuals who frequently interact with the healthcare system, resulting in a sample population that is disproportionately high-income, white, older, and with high comorbidities.^19,21,22^

A major strength of this study was the data source, which comprised a large sample of individuals from 84 healthcare institutions across the U.S.^18^ Similarly, we included a wide range of health characteristics from each patient, allowing us to rigorously adjust for a wide range of potential confounders. The analytic approach that we applied, using Super Learner and Targeted Maximum Likelihood Estimation, was a second strength.^30–34^ The use of highly flexible, semiparametric estimation methods minimized the probability of model misspecification while conducting high-dimensional covariate adjustment, and the use of doubly-robust estimation methods supports the validity of our findings.

## CONCLUSIONS

We found that the prescription of metformin, compared to DPP4i, during acute COVID-19 was associated with lower 12-month mortality among individuals with T2DM. We found modest evidence of a protective relationship between metformin and Long COVID, although the estimation of Long COVID diagnosis was imprecise. These findings support the use of metformin in individuals with T2DM during acute COVID-19 to reduce long-term adverse outcomes.

## Supplementary Material

Supplement 1 Supplemental Material 1. Rationale for secondary analyses.
Supplemental Material 2. Covariates of interest.
Supplemental Material 3. Characteristics of individuals with prediabetes prescribed metformin or levothyroxine during acute COVID-19.
Supplemental Material 4. Characteristics of individuals with PCOS prescribed metformin or levothyroxine during acute COVID-19.
Supplemental Material 5. Relationship between metformin vs. levothyroxine during acute COVID-19 and subsequent 12-month cumulative incidence of Long COVID or death, among individuals with PCOS or prediabetes.
Supplemental Material 6. Discussion of secondary cohort findings.

## Figures and Tables

**Table 1.** Characteristics of individuals with type 2 diabetes prescribed metformin or DPP4i during acute COVID-19.

Characteristic	Value	Metformin Count (Proportion)	DPP4i Count (Proportion)	Total Count (Proportion)
Total		50965 (0.96)	2367 (0.04)	53332 (1)
Sex	Female	26694 (0.52)	1389 (0.59)	28083 (0.53)
Age: mean (SD)		60.23 (12.16)	63.51 (11.96)	60.37 (12.17)
Ethnicity	White Non-Hispanic	29353 (0.58)	1314 (0.56)	30667 (0.58)
	Black or African American Non-Hispanic	8853 (0.17)	491 (0.21)	9344 (0.18)
	Hispanic or Latino Any Race	6761 (0.13)	311 (0.13)	7072 (0.13)
	Unknown	2043 (0.04)	90 (0.04)	2133 (0.04)
	Asian or Pacific Islander Non-Hispanic	3372 (0.07)	137 (0.06)	3509 (0.07)
BMI: mean (SD)		36.32 (8.68)	34.91 (8.25)	36.26 (8.67)
Medical Conditions	Tobacco Smoker	8785 (0.17)	399 (0.17)	9184 (0.17)
	Obese	36375 (0.71)	1545 (0.65)	37920 (0.71)
	Chronic Lung Disease	12683 (0.25)	685 (0.29)	13368 (0.25)
	Hypertension	38033 (0.75)	1737 (0.73)	39770 (0.75)
	Systemic Corticosteroids	23354 (0.46)	1095 (0.46)	24449 (0.46)
	Asthma	8399 (0.16)	395 (0.17)	8794 (0.16)
	Heart Failure	5286 (0.1)	308 (0.13)	5594 (0.1)
	Dementia	1042 (0.02)	62 (0.03)	1104 (0.02)
	Arthritis	1166 (0.02)	66 (0.03)	1232 (0.02)
	Coronary Artery Disease	8806 (0.17)	476 (0.2)	9282 (0.17)
	Cancer	6404 (0.13)	332 (0.14)	6736 (0.13)
	Liver Disease	779 (0.02)	58 (0.02)	837 (0.02)
	Chronic Kidney Disease	4573 (0.09)	353 (0.15)	4926 (0.09)
	Peripheral Vascular Disease	6809 (0.13)	372 (0.16)	7181 (0.13)
	Cerebrovascular Disease	3812 (0.07)	227 (0.1)	4039 (0.08)
	Depression	13827 (0.27)	642 (0.27)	14469 (0.27)
Drugs	Insulin	21255 (0.42)	1214 (0.51)	22469 (0.42)
	ACE Inhibitors	538 (0.01)	33 (0.01)	571 (0.01)
	Statins	1010 (0.02)	58 (0.02)	1068 (0.02)
	Anticoagulants	2084 (0.04)	110 (0.05)	2194 (0.04)
	Aspirin	1548 (0.03)	83 (0.04)	1631 (0.03)
	Torsemide	486 (0.01)	35 (0.01)	521 (0.01)
	Furosemide	6147 (0.12)	392 (0.17)	6539 (0.12)
Measurements	Pre-Prescription HbA1c: mean (SD)	7.65 (1.81)	7.93 (1.88)	7.66 (1.81)
	Serum Creatinine: mean (SD)	0.87 (0.25)	0.95 (0.4)	0.88 (0.26)
	Albumin/Creatinine Ratio: mean (SD)	10.84 (36.45)	13.21 (39.74)	10.95 (36.61)
	Estimated Glomerular Filtration Rate: mean (SD)	77.19 (21.12)	71.27 (23.08)	76.92 (21.25)
Medical Utilization	Visits per Month: mean (SD)	2.63 (3.06)	2.68 (3.39)	2.63 (3.07)

**Table 2.** Unadjusted relationships between metformin vs. DPP4i during acute COVID-19 and subsequent 12-month cumulative incidence of Long COVID or death, among individuals with type 2 diabetes mellitus.

Outcome	Metformin Sample Size	DPP4i Sample Size	Count Metformin Outcomes	Count DPP4i Outcomes	Metformin Unadjusted Risk	DPP4i Unadjusted Risk	Unadjusted RR (95% CI)
Diagnosed Long COVID	50965	2367	635	37	0.01	0.02	0.80 (0.57 to 1.11)
Probable Long COVID	50965	2367	8444	464	0.17	0.20	0.85 (0.78 to 0.92)
Death	50965	2367	1220	128	0.02	0.05	0.44 (0.37 to 0.53)

## Data Availability

All analytic code is available upon request from the N3C Enclave. Access to study data may be requested in the N3C Enclave as “legacy data” pending N3C approval. Access to the N3C Data Enclave is managed by NCATS (https://ncats.nih.gov/research/research-activities/n3c/resources/data-access). Interested researchers must first complete a data use agreement, and next a data use request, in order to access the N3C Data Enclave. Once access is granted, the N3C data use committee must review and approve all use of data and the publication committee must approve all publications involving N3C data.

## Inclusion and ethics statement:

All co-authors and collaborators included in this manuscript have fulfilled the criteria for authorship.
